# Female genital mutilation/cutting in Italy: an enhanced estimation for first generation migrant women based on 2016 survey data

**DOI:** 10.1186/s12889-017-5000-6

**Published:** 2018-01-12

**Authors:** Livia Elisa Ortensi, Patrizia Farina, Els Leye

**Affiliations:** 1 Department of Sociology and Social Research – University of Milan Bicocca, Milan, Italy; 2 International Centre for Reproductive Health – Ghent University, Ghent, Belgium

## Abstract

**Background:**

Migration flows of women from Female Genital Mutilation/Cutting practicing countries have generated a need for data on women potentially affected by Female Genital Mutilation/Cutting. This paper presents enhanced estimates for foreign-born women and asylum seekers in Italy in 2016, with the aim of supporting resource planning and policy making, and advancing the methodological debate on estimation methods.

**Methods:**

The estimates build on the most recent methodological development in Female Genital Mutilation/Cutting direct and indirect estimation for Female Genital Mutilation/Cutting non-practicing countries. Direct estimation of prevalence was performed for 9 communities using the results of the survey FGM-Prev, held in Italy in 2016. Prevalence for communities not involved in the FGM-Prev survey was estimated using to the ‘extrapolation-of-FGM/C countries prevalence data method’ with corrections according to the selection hypothesis.

**Results:**

It is estimated that 60 to 80 thousand foreign-born women aged 15 and over with Female Genital Mutilation/Cutting are present in Italy in 2016. We also estimated the presence of around 11 to 13 thousand cut women aged 15 and over among asylum seekers to Italy in 2014–2016. Due to the long established presence of female migrants from some practicing communities Female Genital Mutilation/Cutting is emerging as an issue also among women aged 60 and over from selected communities. Female Genital Mutilation/Cutting is an additional source of concern for slightly more than 60% of women seeking asylum.

**Conclusions:**

Reliable estimates on Female Genital Mutilation/Cutting at country level are important for evidence-based policy making and service planning. This study suggests that indirect estimations cannot fully replace direct estimations, even if corrections for migrant socioeconomic selection can be implemented to reduce the bias.

## Background

Female genital mutilation/cutting (FGM/C) is an umbrella term for any procedure of modification, partial or total removal or other injury to the female genital organs for non-medical reasons [1]. In 1990 the Inter-African Committee on Traditional Practices Affecting the Health of Women and Children adopted the term 'female genital mutilation'. However, as objections have been raised to this terminology, the more culturally sensitive term ‘female genital cutting’ or the more complete term 'female genital mutilation/cutting (FGM/C)' has become widely used among researchers and international development agencies. FGM/C is recognized internationally as an ‘irreparable, irreversible abuse’, a violation of human rights and an extreme form of discrimination against women [2]. Although it occurs differently across communities, regions and countries, research has underlined some recurrent factors underpinning FGM/C, such as cultural tradition, sexual morals, marriageability, religion, perceived health benefits and male sexual enjoyment [3, 4].

According to the last available estimates for the 31 FGM/C practicing countries in Africa, the Middle East and Asia with available data from national household surveys (30 plus the new country of South Sudan), more than 200 million girls and women alive today have been cut [5]. This estimate does not account for other known FGM/C practicing countries (e.g. Malaysia) nor for women living in western countries as the consequence of female emigration flows from practicing countries to areas where FGM/C was previously unknown such as Europe, Australia or North America [6]. These migration flows have generated a need for data on the prevalence of women potentially affected by FGM/C whose importance has been reaffirmed by the European Parliament in 2014 [7] and the Istanbul Convention of the Council of Europe [8]. Data on FGM/C are a fundamental tool for targeted and evidence-based policy making in western countries [9]. Building on the most recent methodological developments in FGM/C direct and indirect estimation for non-FGM/C practicing countries, this paper presents detailed estimates for foreign-born women and asylum seekers aged 15 and over with FGM/C in Italy in 2016, with the aim of supporting resource planning and policy making.

## Theoretical background

Even though detailed information is needed for the planning and commissioning of health services, as well as to calibrate policies towards the discontinuation of the practice, data on FGM/C are less reliable in the countries of emigration because data based on surveys are usually unavailable. Researchers aiming at estimating the number of women affected by FGM/C must overcome two major challenges: determining a reliable number of women living in emigration (including hypothetically irregular stayers, naturalized women and second generations) and estimating the prevalence among different national groups.

As for the first issue mentioned, examples of the data used as a basis for estimates include labor force surveys [10], population census or survey data on smaller census samples [11, 12], residence permits [13, 14], population’s or foreigners’ registers [15, 16] and data on school attendance [17]. In some studies, data on women requesting political asylum and unaccompanied female minors who were not asylum seekers are also included [18] as citizens from FGM/C practicing countries are usually well-represented among this particular subpopulation. Omission of undocumented migrants, second generation and naturalized citizens causes an underestimation of women with FGM/C. Despite this awareness data covering all women potentially affected or at risk of FGM/C are rarely available.

The second issue is related to prevalence estimation. Most studies build on the application of prevalence data observed in FGM/C practicing countries to women with a practicing country background living abroad [11, 19, 20]. This technique, known as ‘indirect estimation’ or ‘extrapolation-of-FGM/C countries prevalence data method’, is the most systematic, least complex and least costly way of estimating the number of women with FGM/C in Western country settings [21]. However, despite the multiple advantages, the method does not provide a real picture of the phenomenon. Indirect estimation is, in fact, only a combination of FGM/C trends observed in practicing countries and of trends in female migration flows in countries of emigration. The technique has strong methodological limitations as it fails to consider the process of social, geographical and age selection of migrants [22]. Evidence from FGM/C practicing countries indicates that some individual characteristics, such as belonging to younger age cohorts, having higher levels of wealth and education or urban residence, are usually correlated with a lower occurrence of FGM/C [23]. At the same time, the recent surge in studies on contemporary African migration has confirmed the existence of mechanisms of positive selection in international flows from Africa, not least because of the relatively high costs of the journey to Europe [24–27]. The same correlations between migration and good levels of education, middle class status and a young age have also been observed for the subgroup of African female migrants, suggesting a direct impact on the occurrence of FGM/C among immigrants [28–31]. The estimation of FGM/C occurrence among second generation, usually considered less at risk compared to first generations, is also a challenge [32] because the effect of migration on the risk is difficult to assess and can vary according to contexts and communities. For this reason second generations have not been included in this study.

In the field of indirect estimation, recent efforts have been aimed at developing corrections to reduce the bias derived from the application of national estimations to immigrant communities. The work of Exterkate [33] on Dutch data underlines the role of age- and region-specific FGM/C prevalence data to obtain the most realistic approximations of prevalence in immigrant communities. Ortensi and colleagues [22] aimed at obtaining some coefficients in order to correct indirect estimation on the basis of the expected socioeconomic composition of migrants’ flows (the selection hypothesis method). Finally Andro and colleagues [12] corrected indirect estimation on the basis of the women’s ages at arrival and their places of birth.

At the same time, to overcome limitations related to indirect FGM/C prevalence estimation, researchers are increasingly trying to develop methodologies aimed at the direct estimation of FGM/C. The European Directorate-General for Justice has recently funded the Daphne Project FGM-Prev (Grant just/2013/dap/ag/5636) in order to promote a pilot study to test a replicable methodology to estimate FGM/C in Europe [34]. Results from two fieldwork-based studies in Italy and Belgium and the lessons thereby learned have been discussed extensively among experts in order to enhance the possibility of repeating a direct study on FGM/C in a growing number of countries [34].

The current study builds both on direct and indirect methodology aiming at producing an updated and enhanced estimation for Italy in 2016 according to the suggestions of Leye and colleagues [20].

## Methods

### Data

Data on the presence of women in Italy were extracted from the Eurostat database:

- Foreign-born women from practicing countries by five year age group (migr_pop3ctb) as of 1 January 2016
- First time asylum applicants by citizenship, age and sex, annual data (migr_asyappctza) years 2014–2016

These data are available for most EU member states.

Data on the prevalence of FGM/C for women born in Nigeria, Egypt, Eritrea, Senegal, Burkina Faso, Somalia and the Ivory Coast were obtained from the survey conducted in Italy as a part of the Daphne project FGM-Prev. In order to estimate the prevalence of FGM/C in the main communities from FGM/C practicing countries in Italy, a survey was conducted from June to December 2016 covering 1378 women aged 18 and over living in Italy. The methodology developed in the FGM-Prev project is a combination of facility based and respondent driven sampling. The survey was conducted in many Italian cities covering also suburban and mountain areas. The FGM/C status was self-reported by the women interviewed and no physical examination was performed in relation to the survey. The interviews were carried out by a team of female foreign interviewers well acquainted with the issues, and belonging to the communities selected in the sample, who were thus able to translate and formulate questions appropriately. This has been a key factor in facilitating intimate conversation among women trying to reduce voluntary underreporting. We are however aware that these data share most of the limitations expected of surveys on hard-to-reach populations [34] and of survey based on self-reported data on FGM/C status [35].

Prevalence data on FGM/C by 5 year age group were obtained from the latest available DHS [36], MICS (Multiple Indicators Cluster Surveys) [37], PHS (Population and Health Surveys) [38]; or HHS data (Household and Health Survey) [39]. These surveys are the main sources of information about FGM/C in practicing countries [40].

Exceptions are data for Indonesia that were taken from UNICEF [41] and data for South Sudan that were retrieved from Oxfam [42] that estimated prevalence using unpublished data from the Southern Sudan Household Survey of 2010. For Indonesia, the prevalence is available only for girls aged 0–11, and could therefore be considered as a minimum value, while for South Sudan, the prevalence is available for women aged 15–49 absent the detail by 5 year age group. Detailed information on the sources used can be found in column (c) of Table 1.

**Table 1:** Estimated prevalence of FGM/C among foreign-born women from FGM/C practicing countries. Italy 2016

*Country*	*Prevalence of FGM/C among foreign born women and confidence interval* *(a)*	*Correction according to the selection hypothesis* *(b)*	*Most recent national estimation (c)*		
			*%*	*Year*	*Source*
Mali	92.0 (90.6; 93.5)	1.01	91.4	2013	DHS
Sudan	91.5 (90.2; 92.8)	1.04	86.6	2014	MICS
Somalia	89.5 (81.1; 98.0)	Direct estimation	97.9	2006	MICS
Djibouti	83.2 (81.7; 84.6)	0.99	93.1	2006	MICS
Burkina Faso	71.6 (63.7; 79.6)	Direct estimation	75.8	2010	DHS
Guinea	71.0 (68.7; 73.3)	0.95	96.9	2012	DHS
Nigeria	69.8 (56.9; 82.7)	Direct estimation	24.8	2013	DHS
Eritrea	69.8 (58.4; 81.3)	Direct estimation	83.0	2010	Population and Health Survey
Gambia. The	69.6 (67.5;71.8)	0.93	74.9	2013	DHS
Ethiopia	63.7 (61.5;65.8)	0.83	65.2	2016	DHS
Sierra Leone	61.2 (60.5;61.8)	0.87	89.6	2013	DHS
Egypt	60.7 (52.5;68.9)	Direct estimation	87.2	2015	Health Issues Survey (DHS)
Mauritania	52.5 (50.9;54.1)	0.75	69.4	2011	MICS
Indonesia	49.0 (47.1;50.9)	Data not available	49.0	2016	Unicef
Liberia	38.6 (36.6;40.6)	0.68	49.8	2013	DHS
Guinea-Bissau	33.5 (31.4;35.5)	0.74	44.9	2014	MICS
Senegal	27.5 (18;37.1)	Direct estimation	24.7	2014	DHS
Chad	21.0 (19.7;22.3)	0.63	38.4	2014–15	MICS
Kenya	17.5 (16.2;18.9)	0.64	21.0	2014	DHS
Central Afr. Rep.	16.9 (15.4;23.5)	0.62	24.2	2010	MICS
Yemen	15.8 (14.6;17)	0.77	18.5	2013	DHS
Côte d’Ivoire	10.7 (2.4;19)	Direct estimation	38.2	2011–12	DHS
Tanzania	7.2 (6.3;8.1)	0.39	14.6	2010	DHS
Iraq	4.8 (4.4;5.2)	0.52	8.1	2011	DHS
Benin	3.8 (3.3;4.2)	0.30	9.2	2014	MICS
Togo	2.6 (1.9;3.2)	0.45	4.7	2013–2014	DHS
Uganda	1.6 (0.8;2.4)	1.05	1.4	2011	DHS
Ghana	1.4 (1;1.7)	0.32	3.8	2011	MICS
Niger	1.1 (0.7;1.4)	0.55	2.0	2012	DHS
Cameroon	0.6 (0.3;1)	0.46	1.4	2004	DHS
South Sudan	1.4 (0.04; 2.4)	Data not available	1.4	2010	Southern Sudan Household Survey

Source: Authors’ elaboration from FGM-Prev Survey and DHS/MICS/PHS/HHS surveys

### Method

Prevalence for communities *i* included in the FGM-Prev survey (Nigeria, Egypt, Eritrea, Senegal, Burkina Faso, Somalia and Ivory Coast) was obtained directly. The subsample for each community was not enough to ensure the possibility of calculating a 5 five year age group prevalence, so, for each community, we calculated the proportion of cut women (${p}_j^i$) aged *j* = 18 − 34 and *j* = 35+ . This passage was implemented in order to account for broader age differences in FGM/C prevalence and obtain a more accurate estimation compared to that based on the overall prevalence of women aged 18 and over. As women aged 15–17 were not included in the survey for ethical reasons (minors), we applied to this group the 18–34 age prevalence.

For countries *i* included in the survey the number of women aged 15 and above with FGM/C was calculated as

1 $$ \overline{W^i}=\sum \limits_{j=15-34,35+}\left({p}_j^i\right)\left({W}_j\right) $$

Where *W*_*j*_ is the number of women aged *j* and born in the country *i* in Italy as of 1 January 2016 according to Eurostat data.

Indirect estimation was calculated starting from the last available prevalence data by 5 five year age group for each community *k* lacking a direct estimation on the basis of the FGM-Prev survey. Before applying DHS/MICS prevalence data to the female population from practicing countries in Italy, we applied the procedure of FGM/C prevalence correction for immigrant communities according to the selection hypothesis (the detailed procedure is explained in [22, 43]. The method is based on the theoretical assumption that migration is a selective process and is aimed at reducing the bias arising from the correlation observed in practicing countries of FGM/C occurrence with wealth, education and urban residence [23].

The selection hypothesis was implemented excluding the correction for age as the real 5 years-age structure for each community is known in this study.

So for each practicing country *k* we computed the correction

2 $$ {s}_k= mean\left(\ \frac{m_{urb,k}}{m_k},\frac{m_{hedu,k}}{m_k},\frac{m_{hw,k}}{m_k}\right) $$

according to the most recent DHS/MICS/PHS/HHS data available.

Where:

*m*_*urb*, *k*_ is the prevalence of FGM/C among women settled in urban areas in the country *k.*

*m*_*hedu*, *k*_ is the prevalence of FGM/C among women with a higher level of education in the country *k.*

*m*_*hw*, *k*_ is the prevalence of FGM/C among women belonging to the highest wealth quintile in the country *k.*

*m*_*k*_ is the prevalence of FGM/C among all women in the country *k.*

The use of an unweighted mean is due to the fact that we miss detailed information about the composition of past flows of migrants by education level, wealth quintile of the family of origin or place of birth (urban/rural). The correction is expected to get the order of magnitude and the direction of the difference between national prevalence and overseas community prevalence for communities where other factors correlated with FGM/C prevalence (e.g. a strong geographical or a strong ethnic selection) are not preponderant. The coefficients applied for each community *k* are reported in column (b) of Table 1. The estimation $\left({p}_j^k\right)$ corrected on the basis of the selection hypothesis is obtained by simply applying the set of coefficient *s*_*k*_ to the baseline estimation of the number of expected women with FGM/C from each practicing country *k* ${P}_{fgm/c}^k$

3 $$ {p}_j^k=\left({P}_{fgm/c}^k\right){s}_k $$

For communities *k* not included in the survey, the number of women aged 15 and above with FGM/C was calculated as:

4 $$ \overline{W^k}={\sum}_{j=x-\left(x+4\right)}\left({p}_j^k\right)\left({W}_j\right)\;\mathrm{where}\;\mathrm{x}-\left(\mathrm{x}+4\right)=15-19,20-24,.\dots 65+\mathrm{is}\kern0.17em \mathrm{the}\;5\ \mathrm{years}\  \mathrm{group} $$

and *W*_*j*_ is the number of women aged *j =* x-(x + 4) and born in the country *k* in Italy as of 1 January 2016 according to Eurostat migr_asyappctza data.

The final number of estimated foreign born women with FGM/C is the simple sum of the direct and indirect estimations

5 $$ \overline{W}={\sum}_i{W}^i+{\sum}_k{W}^k $$

Each estimated prevalence was provided with a confidence interval.

We repeated the same procedure for data on first time asylum applicants in the period 2014–2016. In the application of indirect estimation to first time asylum application data, prevalence based on two age groups (15–34, 35+) was applied due to the structure of Eurostat data.

According to latest population data, communities selected in FGM-Prev survey account for 66% of the foreign-born women from practicing countries in Italy in 2016.

## Results

For countries with small differences at the national level in FGM/C prevalence in terms of education, wealth index and urban setting [23], the prevalence estimated applying the extrapolation-of-FGM/C countries prevalence data method with corrections is substantially unchanged for Italy compared to the national level. This is the case for Mali, Uganda, Sudan or Djibouti. On the contrary, for the other communities such as Benin, Tanzania, Togo or Cameroon the expected prevalence in emigration was substantially reduced compared to the country estimation.

The proportion of women with FGM/C among communities varies significantly, ranging from a group of very high prevalence countries (>80%) such as Somalia, Sudan, Mali and Djibouti to a group characterized by a very low prevalence (<2%) such as Uganda, Ghana, Niger, Cameroon and South Sudan (Table 1).

As a consequence of the estimated prevalence rates, 60 to 80 thousand foreign-born women aged 15 and over with FGM/C are present in Italy in 2016. (Table 2).

**Table 2:** Number of foreign-born women and estimated cut women from FGM/C practicing countries. Italy 2016

*Country*	*Foreign-born* *women*	*Expected foreign-born women with FGM/C and confidence interval*
Nigeria	31,292	21,847 (17,809;25,884)
Egypt	27,755	16,856 (14,578;19,135)
Senegal	19,256	5301 (3457;7144)
Ghana	16,843	231 (176;286)
Ethiopia	15,534	9891 (9561;10,221)
Côte d’Ivoire	10,259	1095 (243;1948)
Eritrea	6009	4195 (3507;4883)
Cameroon	5698	36 (16;56)
Somalia	4612	4128 (3738;4519)
Burkina Faso	3172	2272 (2019;2524)
Indonesia	2574	1261 (1212;1311)
Kenya	2152	377 (348;406)
Togo	1713	44 (33;54)
Guinea	1163	825 (799;852)
Iraq	960	46 (43;50)
Benin	950	36 (32;40)
Tanzania	931	67 (59;76)
Sudan	621	568 (560;576)
Uganda	564	9 (5;14)
Sierra Leone	527	322 (319;326)
Mali	504	464 (457;471)
Liberia	307	119 (112;125)
Gambia, The	302	210 (204;217)
Guinea-Bissau	242	81 (76;86)
Niger	209	2 (1;3)
Mauritania	159	84 (81;86)
Chad	117	25 (23;26)
Yemen	113	18 (17;19)
Central African Republic	93	16 (14;22)
Djibouti	52	43 (42;44)
South Sudan	13	0 (0;0)
Total	154,694	70,469 (59,540; 81,404)

Source: Authors’ elaboration from FGM-Prev Survey DHS/MICS/PHS/HHS surveys and Eurostat data

Given the combination of large communities and high FGM/C prevalence rates, Nigerian and Egyptian women made up more than half of the foreign-born women with FGM/C. Another 14% of cut women was born in Ethiopia and the 7% was born in Senegal.

The composition of cut women by age is also the result of historic female flows from Africa to Italy. Women from Eritrea, Somalia and Ethiopia were among the first to migrate to Italy, forerunning the mass immigration that started from the beginning of the 90s [44]. The age structures of foreign-born women from Eritrea, Somalia and Ethiopia therefore differ from those of other practicing countries, showing a high proportion of women aged 65 and over (respectively 47.6% among Eritrean, 45.3% among Ethiopians and 23.6% among Somalis compared to an overall proportion of 8.7%) most of them cut (Table 3). The presence of around 18,000 women aged 60 and above with FGM/C is a new issue for health services dedicate to elderly in Italy.

**Table 3:** Number of foreign-born women and estimated cut women from FGM/C practicing countries by 5 year age groups. Italy 2016^a^

Age groups	Total women	Women with FGM/C (medium variant)	FGM/C prevalence	First three communities by age
From 15 to 19 years	7702	2761	35.9	Egypt, Nigeria, Senegal
From 20 to 24 years	10,257	4018	39.2	Nigeria, Egypt, Senegal
From 25 to 29 years	18,615	8222	44.2	Nigeria, Egypt, Senegal
From 30 to 34 years	23,553	10,711	45.5	Nigeria, Egypt, Senegal
From 35 to 39 years	25,071	11,140	44.4	Nigeria, Egypt, Senegal
From 40 to 44 years	21,269	9177	43.1	Nigeria, Egypt, Senegal
From 45 to 49 years	15,220	6464	42.5	Nigeria, Egypt, Ethiopia
From 50 to 54 years	9112	4070	44.7	Nigeria, Ethiopia, Egypt
From 55 to 59 years	5797	2988	51.5	Ethiopia, Egypt, Nigeria
From 60 to 64 years	4555	2720	59.7	Ethiopia, Egypt, Eritrea
65 years or over	13,543	9249	68.3	Ethiopia, Egypt, Eritrea
Total	154,694	71,522	46.2	Nigeria, Egypt, Ethiopia

^a^According to the medium variant estimate, column (a) in Table 1

Source: Authors’ elaboration from FGM-Prev Survey, DHS/MICS/PHS/HHS surveys and Eurostat data

We also estimate the presence of around 11 to 13 thousand cut women among asylum seekers aged 15 and over to Italy during 2014–2016 (Table 4). The presence of around 60% of cut women among such a vulnerable population requires further attention in terms of assistance at their reception to the country. Of course, we are aware that some of these women especially rejected asylum applicants may have left Italy. Nigerians women are largely predominant among cut asylum seekers (78.6%). Other groups with an expected large numbers of cut women are from Eritrea and Somalia (respectively 7.1% and 6.5% of all expected cut asylum seekers).

**Table 4:** Estimated number of women with FGM/C and prevalence of FGM/C among asylum applicants. Italy 2014–2016

Country	Asylum applications 2014–2016	Expected asylum applicants with FGM/C and confidence interval	Prevalence of FGM/C among asylum applicants and confidence interval
Nigeria	12,305	9928 (9078;10,778)	80.7 (73.8; 87.6)
Egypt	75	42 (36;48)	55.7(47.5; 63.8)
Somalia	950	816 (706;926)	85.9 (74.3;97.5)
Eritrea	1960	899 (666;1133)	45.9 (34;57.8)
Ethiopia	175	93 (89;96)	52.9 (50.8; 55.0)
Senegal	275	75 (45;106)	27.4 (16.3; 38.5)
Burkina Faso	40	27 (24;30)	67.7 (59.4; 76.1)
Côte d’Ivoire	1210	145 (34;257)	12.0 (2.8; 21.2)
Guinea	130	92 (87;94)	71.1 (67.2;72.7)
Sierra Leone	90	66 (66;67)	73.9(72.8;74.9)
Sudan	30	27 (26;27)	89.6 (88.3;90.9)
Mali	195	179 (176;181)	91.8 (90.5;93.1)
Indonesia	0	0 (0;0)	0.0 (0.0;0.0)
Ghana	275	2 (2;3)	0.8 (0.6;1.1)
Mauritania	10	5 (5;5)	52.4 (50.8;54)
Gambia, The	280	195 (190;200)	69.8 (68;71.6)
Kenya	60	9 (9;10)	15.5 (14.8;16.7)
Guinea-Bissau	5	5 (5;5)	91.8 (90.5;93.1)
Liberia	15	4 (4;4)	26.6 (25;28.2)
Togo	45	0 (0;1)	1.1(0.8; 1.4)
Benin	20	0 (0;0)	2.2(1.9; 2.5)
Iraq	220	7 (7;8)	3.4 (3.1; 3.6)
Chad	5	1 (1;1)	23.1 (22.6;19.2)
Tanzania	5	0 (0;0)	0 (0.0; 0.0)
Yemen	10	2 (1;2)	15.0 (14;16.1)
Cameroon	665	3 (1;4)	0.4 (0.2;0.6)
Uganda	25	0 (0;0)	0.0 (0;0)
Niger	20	0 (0;0)	0.0 (0;0)
Central African Republic	15	2 (2;3)	12.3 (11.3;17.6)
Djibouti	0	0 (0;0)	0.0 (0;0)
South Sudan	0	0 (0;0)	0.0 (0;0)
Total	19,110	12,626 (11,260;13,990)	66.1 (58.9;73.2)

Source: Authors’ elaboration from FGM-Prev Survey DHS/MICS/PHS/HHS surveys and Eurostat data

## Discussion

**Fig. 1:**
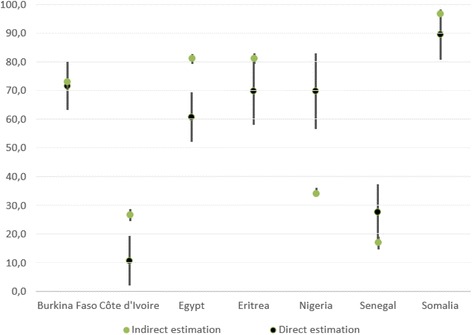
Comparison between direct and indirect estimation of prevalence for women aged 15 and over for selected FGM/C practicing countries. Italy 2016. Source: Authors’ elaboration from FGM-Prev Survey and DHS/MICS/PHS/HHS surveys

Reliable data on women with FGM/C are needed to guide effective policies and interventions on health care and prevention. Studies on this topic are key to estimate and allocate the resources to meet the actual needs of women who are in potential need of health care for related physical and psychological complications [19]. The use of dedicated surveys instead of indirect estimations is of particular importance because the prevalence found among immigrants may be different from that estimated in the country of origin. Our study shows that in the case of Burkina Faso, Eritrea, Senegal and Somalia, the indirect estimations with corrections according to the selection hypothesis fall in the confidence interval of the direct estimation, although they are sometimes close to the extreme bound (Fig. 1). In the other cases, the correction based on the selection hypothesis (a reduction for Egypt and Ivory Coast and an increase in the case of Nigeria) predicts correctly the direction of the expected variation as compared with the country of origin. However, the intensity of the variation is underestimated, confirming previous results [22].

The underestimation of the phenomenon is particularly problematic in the case of Nigeria, one of the main communities affected by FGM/C. The high prevalence observed among Nigerian immigrants is due to the strong geographic selection of flows to Italy. Most flows from Nigeria to Italy are from the Edo State, but some women are also from the nearby areas of Delta State, Lagos State, Ogun State and Anambra State [45]. All these areas are characterized by a higher FGM/C prevalence rate than the overall country [46]. The high FGM/C prevalence among Nigerian women is also a consequence of selection: in Nigeria an association between FGM/C occurrence and positive socioeconomic selection, uncommon in most of other FGM/C practicing countries, is in fact observed [46]. When we strongly underestimate the occurrence of FGM/C in one of the main communities settled in a country we also underestimate the magnitude of resources needed for care and prevention. The high occurrence of FGM/C among Nigerian women in Italy is also of particular concern because cases of trafficking and forced prostitution have been frequently reported by social workers for migrants in this community. The high occurrence of FGM/C is therefore an additional concern in a community characterized by a high degree of vulnerability [45].

We also underline that second generation girls and women are not included in this study, because we are aware that different techniques of estimation are required to address this particular subpopulation and detailed data for Italy are unavailable [32]. Readers and policy makers should be therefore aware that our estimation lacks the detail for girls at risk or cut aged 0–14, which are an additional source of concern.

Given the role of Italy as a major receiver of asylum applications, the high number of expected women with FGM/C is an additional source of concern. We know that migration along the central Mediterranean route is particularly risky for women: the rates of trafficking for sexual exploitation are high and increasing, torture, slavery and sexual violence are often experienced by asylum seekers before they reach the Italian shores [47]. FGM/C is an additional source of concern for women seeking asylum.

It is not possible to compare directly our estimates on legally present foreign born women aged 15 and over to previous data for Italy [11, 48]. The work of Farina and colleagues [48], who estimate the presence of 57,000 foreign girls with FGM/C in 2010, builds on a methodological approach to the estimation of FGM/C prevalence similar to our study but the prevalence is applied to foreign women aged 15–49 including also undocumented migrants. The work of Van Baelen and colleagues [11] who estimate the presence of 59,700 legally present foreign born women in 2011 is based on an extrapolation from age-specific FGM/C prevalence rates without corrections on data census data on girls and women aged 10 and over. This work is therefore based on a different method for the estimation of prevalence and on a different data source and age span of girls and women included in the study.

The difference in the number of estimated women with FGM/C is due to the overall growth in the number of women from FGM/C practicing countries between 2010/2011 and 2016, to different age spans considered, different classification and legal status of the women involved (foreign born vs. foreign, only legal migrants vs. undocumented migrants) and different methods of prevalence estimation.

Despite the difference in data and methods with previous studies we can assume that the number of women affected by FGM/C in Italy is rising, while projections for Italy suggest that around 65,000 women with FGM/C will migrate to Italy between 2016 and 2030 due to economic driven factors [43].

## Conclusion

Reliable estimates on FGM/C at country level are important for evidence-based policy making and service planning. This study presents an example of enhanced estimation of women with FGM/C born in practicing countries, based on the results of a dedicated survey covering the most important communities in Italy. In this study, the bias arising from the application of the extrapolation-of-FGM/C countries prevalence data method is limited to smaller communities and corrections according to the selection hypothesis have been implemented. We aspired to estimate the number of FGM/C cases in two groups with different policy implications: foreign born women and asylum applicants. Our estimate suggests that around 60 to 80 thousand foreign-born women aged 15 and over with FGM/C are present in Italy in 2016. We also estimated the presence of around 11 to 13 thousand cut women aged 15 and over among asylum seekers to Italy in 2014–2016 who may be in particular need of assistance. Second generations girls who may be at risk of undergoing FGM/C are not included in this estimation; further studies are needed to assess the risk in this particular subgroup of women and children.

## Abbreviation

DHS: Demographic and Health Surveys
FGM/C: Female genital mutilation/cutting
HHS: Household and Health Survey
MICS: Multiple Indicators Cluster Surveys
PHS: Population and Health Surveys
